# Oscillometry of the respiratory system in Parkinson's disease: physiological changes and diagnostic use

**DOI:** 10.1186/s12890-023-02716-w

**Published:** 2023-10-26

**Authors:** Bruno Tavares Caldas, Fernando Carlos Vetromille Ribeiro, João Santos Pereira, Wilma Costa Souza, Agnaldo José Lopes, Pedro Lopes de Melo

**Affiliations:** 1 Department of Physiological Sciences, Biomedical Instrumentation Laboratory, State University of Rio de Janeiro, Rio de Janeiro, Brazil; 2 Department of Neurology, Pedro Ernesto University Hospital, State University of Rio de Janeiro, Rio de Janeiro, Brazil; 3 Carioca Parkinson Association, Municipal Rehabilitation Center, Rio de Janeiro, Brazil; 4 Department of Pulmonology, Respiratory Function Testing Laboratory, State University of Rio de Janeiro, Rio de Janeiro, Brazil

**Keywords:** Forced oscillation technique, Respiratory impedance, Diagnostic of respiratory diseases, Smoking

## Abstract

**Background:**

Lung function analysis in Parkinson's disease (PD) is often difficult due to the demand for adequate forced expiratory maneuvers. Respiratory oscillometry exams require onlyquiet tidal breathing and provide a detailed analysis of respiratory mechanics. We hypothesized that oscillometry would simplify the diagnosis of respiratory abnormalitiesin PD and improve our knowledge about the pathophysiological changes in these patients.

**Materials and methods:**

This observational study includes 20 controls and 47 individuals with PD divided into three groups (Hoehn and Yahr Scale 1–1.5; H&Y scale 2–3 and PD smokers).The diagnostic accuracy was evaluated by investigating the area under the receiver operating characteristic curve (AUC).

**Results:**

Initial stages are related to increased peripheral resistance (Rp; *p* = 0.001). In more advanced stages, a restrictive pattern is added, reflected by reductions in dynamic compliance (*p* < 0.05) and increase in resonance frequency (Fr; *p* < 0.001). Smoking PD patients presented increased Rp (*p* < 0.001) and Fr (*p* < 0.01). PD does not introduce changes in the central airways. Oscillometric changes were correlated with respiratory muscle weakness (R = 0.37, *p* = 0.02). Rp showed adequate accuracy in the detection of early respiratory abnormalities (AUC = 0.858), while in more advanced stages, Fr showed high diagnostic accuracy (AUC = 0.948). The best parameter to identify changes in smoking patients was Rp (AUC = 0.896).

**Conclusion:**

The initial stages of PD are related to a reduction in ventilation homogeneity associated with changes in peripheral airways. More advanced stages also include a restrictive ventilatory pattern. These changes were correlated with respiratory muscle weakness and were observed in mild and moderate stages of PD in smokers and non-smokers. Oscillometry may adequately identify respiratory changes in the early stages of PD and obtain high diagnostic accuracy in more advanced stages of the disease.

**Supplementary Information:**

The online version contains supplementary material available at 10.1186/s12890-023-02716-w.

## Background

Parkinson's Disease (PD) is the second most common neurodegenerative disease, affecting 6.1 million globally [1]. It is caused by the death of dopaminergic neurons in the substantia nigra, causing motor symptoms, such as bradykinesia, joint stiffness, and resting tremor, in addition to non-motor symptoms, such as respiratory dysfunctions associated with reduced quality of life [2, 3]. Furthermore, there is an inverse relationship between the incidence of PD and smoking [4–7].

The primary cause of mortality in PD is related to respiratory disorders, including respiratory failure and pneumonia [8–11]. Currently, there are descriptions related to obstructive dysfunction [12–14], restrictive dysfunction [13, 14], and respiratory muscle weakness [13]. However, although they may present such dysfunctions, it is common for them not to manifest dyspnea [15], contributing to the late diagnosis of respiratory disease, when clinical control is more complex [2, 12].

Respiratory oscillometry aims to evaluate the mechanical properties of the respiratory system from the analysis of respiratory impedance characterized by reactance and resistance [16]. The examinations are carried out non-invasively utilizing externally-applied pressure fluctuations under conditions of spontaneous ventilation. Unlike spirometry, these exams require little cooperation [17–19], because it does not require muscle force and coordination to generate the deep inspiration involved in traditional pulmonary function exams. This characteristic may be an essential advantage for patients with PD, in which traditional analysis may be difficult due the presence of tremor and thoracic dyskinesia.

Currently, respiratory oscillometry represents the state-of-the-art in the assessment of pulmonary function for research purpose [18], having been used by our group [20–24] and by other researchers [25–27] to increase our understanding of the pathophysiology of various diseases. Although this method can simplify the exams and enhance the understanding of the pathophysiology of respiratory disorders in individuals with PD, only two preliminary studies performed this analysis [28, 29].

In this context, the main aims of the present study were: (1) to improve our understanding of the pathophysiological changes in individuals with PDusing oscillometry and (2) to evaluate the diagnostic potential of this method, identifying the best parameters for the diagnosis of respiratory changes in these patients.

## Materials and methods

### Study design and ethics

This research is an observational, cross-sectional study whose protocol follows the guidelines of the declaration of Helsinki. The study was approved by the ethics and research committee of Pedro Ernesto University Hospital (456/1997-CEP/HUPE) and registered in the Brazilian Clinical Research Platform (01486312.6.0000.5259, 09/11/2017). The volunteers consented to the study by signing the free and informed consent form. Clinical evaluation, manovacuometry, spirometry, and respiratory oscillometry were performed at the Biomedical Instrumentation Laboratory of the State University of Rio de Janeiro (LIB-UERJ).

### Study population

The study population consisted of smokers and non-smokers with PD from the Movement Disorders outpatient clinic of the Neurology outpatient clinic at HUPE-UERJ and the Parkinson Carioca Association at Nise da Silveira Institute. These patients were evaluated from January 2019 to February 2020. The diagnosis of PD is essentially clinical and is based on motor alterations such as bradykinesia, rigidity, and resting tremor, according to the criteria of Hughes et al. [30]. Patients were divided into three groups, the first with active smokers and ex-smokers (PGtab) and two non-smokers groups, according to the Modified Hoehn and Yahr Scale [31], Parkinson group 1–1.5 (PG1–1.5), and Parkinson's group 2–3 (PG2–3). The amount of tobacco and duration of smoking was quantified using the number of pack-years, calculated by multiplying the average number of packs (20 cigarettes) consumed daily by the number of years of smoking [32]. A control group (CG) was also evaluated, formed by never smoking volunteers over 18 years of age, with a spirometric test within the normal range.

For all groups, the following exclusion criteria were considered: inability to perform spirometry or respiratory oscillometry; body mass index above 35 kg/m^2^; other pulmonary, cardiovascular, and neurological diseases besides PD; Respiratory infections in the last thirty days to the examination; furthermore, only for Parkinson's groups: PD grade 5 stratified by the Modified Hoehn and Yahr scale [31]; Cognitive deficit assessed by the Mini-Mental State Examination [33, 34].All studied volunteers have no history of SARS-CoV-2 infection.

### Traditional exams

Respiratory muscle strength was evaluated with the Ventcare analog manovacuometer, measuring the Maximum Inspiratory Pressure (MIP) and Maximum Expiratory Pressure (MEP). The values found were compared with predicted values [35]. Spirometric measurements were performed in a computerized system (nSpire Health, Inc., 1830 Lefthand Circle, Longmont, CO 80501). The following parameters were evaluated: forced vital capacity (FVC), forced expiratory volume in one second (FEV_1_), FEV_1_/FVC ratio, forced expiratory flow between 25–75 of vital capacity (FEF 25–75%), and peak expiratory flow (PEF). The values found were compared with those predicted [36]. Both exams followed international guidelines [37, 38].

### Respiratory oscillometry

The used instrument was based on pseudorandom, forced oscillationrespiratory oscillometry, and wasdescribed in detail elsewhere [39]. Briefly, the system used pressure oscillations in the frequency range of 4–32 Hz, with amplitude of approximately 2 cmH_2_O. Pressures were produced by a loudspeaker and were coupled to the respiratory system through a mouthpiece for individual use, following international standards [17]. During the exams, the individuals remained seated, with the head in a neutral position and using a nose clip, breathing calmly through a mouthpiece, and firmly supporting the cheeks and lower part of the chin to minimize the shunt effect of the upper airways [17, 20, 24, 40].

The interpretation of oscillometric measurements was based in recent international consensus [17, 25]. Respiratory reactance includes the effects of respiratory compliance and inertance [17]. Compliance reflects the rigidity of the respiratory system, including the compressibility of gas in the airways and alveoli and the elastic forces of the rib cage and lung tissue [17, 41]. The lower the compliance, the more negative the reactance. Inertance, in turn, is associated with air acceleration in the airways.

The reactance curves are interpreted using the average reactance (Xm), calculated through the average of the reactance values in the frequency range between 4 to 32 Hz, being related to the inhomogeneity of the respiratory system [23, 24, 42, 43]. When there is a cancellation between the effects of compliance and inertance, it occurs at the resonance frequency (Fr), linked to changes in the homogeneity of elastic properties [24, 42]. Dynamic compliance (Cdyn) includes compliance with all components involved in ventilation. Cdyn was calculated using the reactance at 4 Hz (Cdyn = 1/2πfX4). The reactance is also interpreted using the area under the reactance curve (Ax). This parameter was recently identified as suitable for predicting the prognosis of patients with COPD [44].

Resistance represents all elements that oppose airflow [17, 41]. The resistance curves are interpreted using the resistance at 4 Hz (R4), related to the total respiratory resistance, and at 20 Hz (R20), which is linked to the most central airways. We also evaluated the frequency dependence of the resistance represented by the difference between R4 and R20 (R4-R20) [20], associated with ventilation homogeneity [24, 45].

**Fig. 1:**
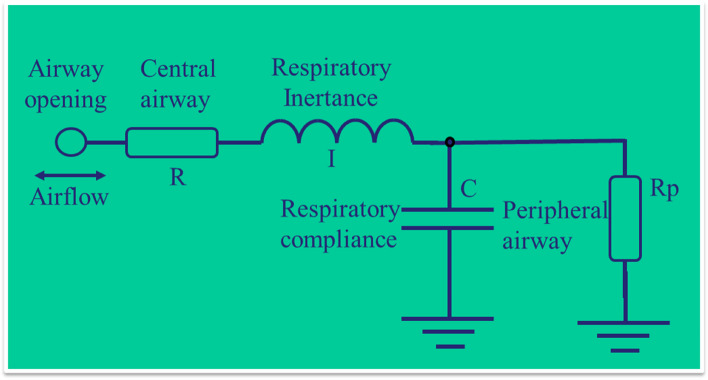
Respiratory model used to interpret oscillometry results describing the effect of the central airway (R), respiratory inertance (I) and compliance (C), and peripheral airway (Rp)

More details on respiratory changes can be obtained using respiratory system models. These models can help investigate the pathophysiology and diagnosis of the disease [20–24]. In the present study, we used the extended RIC model (eRIC, Fig. 1), which allows us to obtain information separating the resistance relative to the central airways (R) from the peripheral airways resistance (Rp), as well as to evaluate the total resistance (Rt = R + Rp). Additionally, information on respiratory inertance (I) and compliance (C) is also obtained [20, 24, 45]. This model was recently used to investigate the short‑term effect of autogenic drainage on peripheral resistance in childhood cystic fibrosis disease [26]and small airway dysfunction in preschool asthma [46].

### Statistical analysis

Initially, the distribution characteristics of the samples were evaluated using the Shapiro–Wilk normality test. The effect of PD stages was investigated using one-way ANOVA with Tukey’s test in the normally distributed data; conversely, a non-parametric analysis (Kruskal–Wallis with a Dunn's Test for post-hoc analysis) was performed for the non-normally distributed data. The effect of smoking was investigated comparing the PGtab group with each one of the other studied groups. This was done using independent Test-t when the sample presented a parametric characteristic and the Mann–Whitney Test when it was not parametric. Manovacuometry analysis was performed by a comparison with predicted values. The value of *p* < 0.05 was used to consider statistically significant differences. Correlations were studied using Pearson's correlation coefficient in the presence of normal distributions, while Spearman's correlation was used in non-normal distributions. These analyzes were conducted in measured values and performed using the OriginPro 2023.

The required sample size was calculated based on the results of a pilot study with a smaller number of patients [47]. Using MedCalc® software (MedCalc Software, Mariakerke, Belgium) and assuming 10% type I and type II errors in the group PG1–1.5, the minimum sample size was 13 volunteers in each group (controls and patients).A similar analysis assuming 1% type I and type II errors in the PG2–3resulted in a minimum sample size of 19 volunteers in each group.

The diagnostic potential of the oscillometric parameters in individuals with PD was evaluated using Receiver Operating Characteristic (ROC) curves. The discrimination between each studied PD group and healthy subjects was evaluated. The sensitivity and specificity of respiratory oscillometric parameters and diagnostic accuracy through the area under the ROC curve (AUC) were evaluated. It was considered an adequate diagnostic performance when the AUC value is > 0.80, while between 0.90 and 1.00, the value indicates high diagnostic accuracy [48, 49]. The software used for this calculation was the MedCalc® program (*Medicalc Software, Belgium*).

## Results

As shown in Table 1, we enrolled a group of 67 participants (20 controls and 47 patients). Regarding the anthropometric variables, no significant differences were observed. A significant reduction was observed when comparing PG1–1.5 with controls in FVC(%) and PEF(%), in addition to a significant reduction in FVC(L) (*p* < 0.05) in PG2-3 compared to CG.

**Table 1:** Biometric and spirometric characteristics of studied groups

	Control (*n* = 20)	Parkinson 1–1.5 (*n* = 13)	Parkinson 2–3 (*n* = 21)	Parkinson smoker (*n* = 13)
Age (years)	66.0 ± 9.8	65.2 ± 11.8	64.1 ± 9.1	66.6 ± 7.4
Body mass (kg)	70.6 ± 12.6	72.3 ± 10.0	66.1 ± 14.9	74.8 ± 13.1
Height (cm)	167.9 ± 10.1	168.9 ± 9.8	162.5 ± 11.1	168.9 ± 5.8
BMI (kg/m^2^)	24.9 ± 2.5	25.4 ± 3.7	24.7 ± 3.8	26.1 ± 3.4
FEV_1_ (L)	2.8 ± 0.7	2.6 ± 0.6	2.4 ± 0.7	2.7 ± 0.6
FEV_1_ (%)	93.6 ± 12.1	86.4 ± 11.6	88.5 ± 15.8	87.2 ± 15.5
FVC (L)	3.7 ± 1.0	3.3 ± 0.8	3.1 ± 0.8^*^	3.5 ± 0.8
FVC (%)	96.7 ± 11.5	86.5 ± 9.1^**^	89.2 ± 15.1	87.7 ± 12.8
FEV_1_/FVC	75.7 ± 5.1	77.6 ± 7.4	78.3 ± 6.4	76.9 ± 5.3
FEV_1_/FVC (%)	90.1 ± 5.9	99.9 ± 8.1	99.3 ± 7.4	99.3 ± 6.5
PEF (L/s)	7.4 ± 2.3	6.1 ± 2.6	6.2 ± 2.7	7.6 ± 2.2
PEF (%)	80.5 ± 23.9	64.4 ± 18.4^*^	70.6 ± 25.4	76.4 ± 19.1
FEF_25-75%_ (L/s)	2.4 ± 0.8	2.4 ± 1.1	2.4 ± 0.9	2.5 ± 1.1
FEF_25-75%_ (%)	89.1 ± 27.1	93.4 ± 32.5	94.3 ± 29.9	92.3 ± 37.4

*BMI* Body mass index, *%* percentage of the predicted value, *FEV*_*1*_ Forced expiratory volume in the first second, *FVC* Forced vital capacity, *FEV*_*1*_*/FVC* The ratio of FEV_1_ to FVC, *n* number of patients evaluated, *PEF* Peak expiratory flow, *FEF 25–75* Forced expiratory flow at 25 and 75%, *Ns* non significance. *P* value

^*^p < 0.05

^**^p < 0.01

**Fig. 2:**
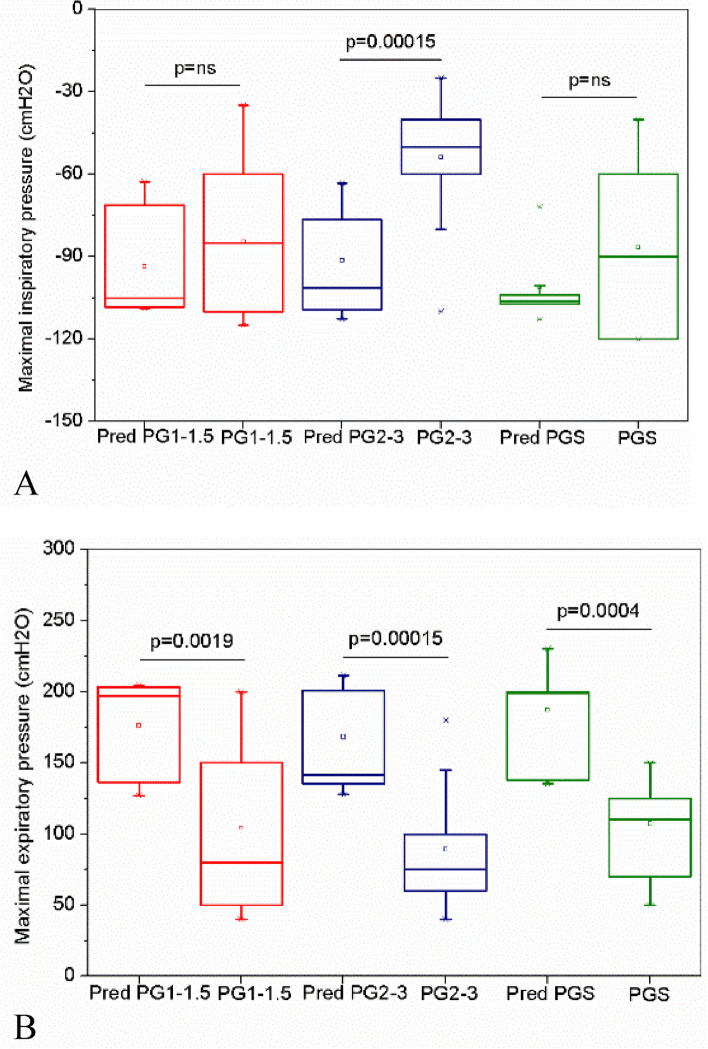
Respiratory muscle strength in the studied groups. Maximal inspiratory **A** and expiratory **B** pressures describing the predicted (Pred) and measured values in groupswith Parkinson1-1.5 (PG1-1.5), 2–3 (PG2-3) and smoking patients (PGS)

Considering the respiratory pressures, a significant reduction in MIP was observed only in PG2-3, while MEP showed significant reductions in all studied groups (Fig. 2).

**Fig. 3:**
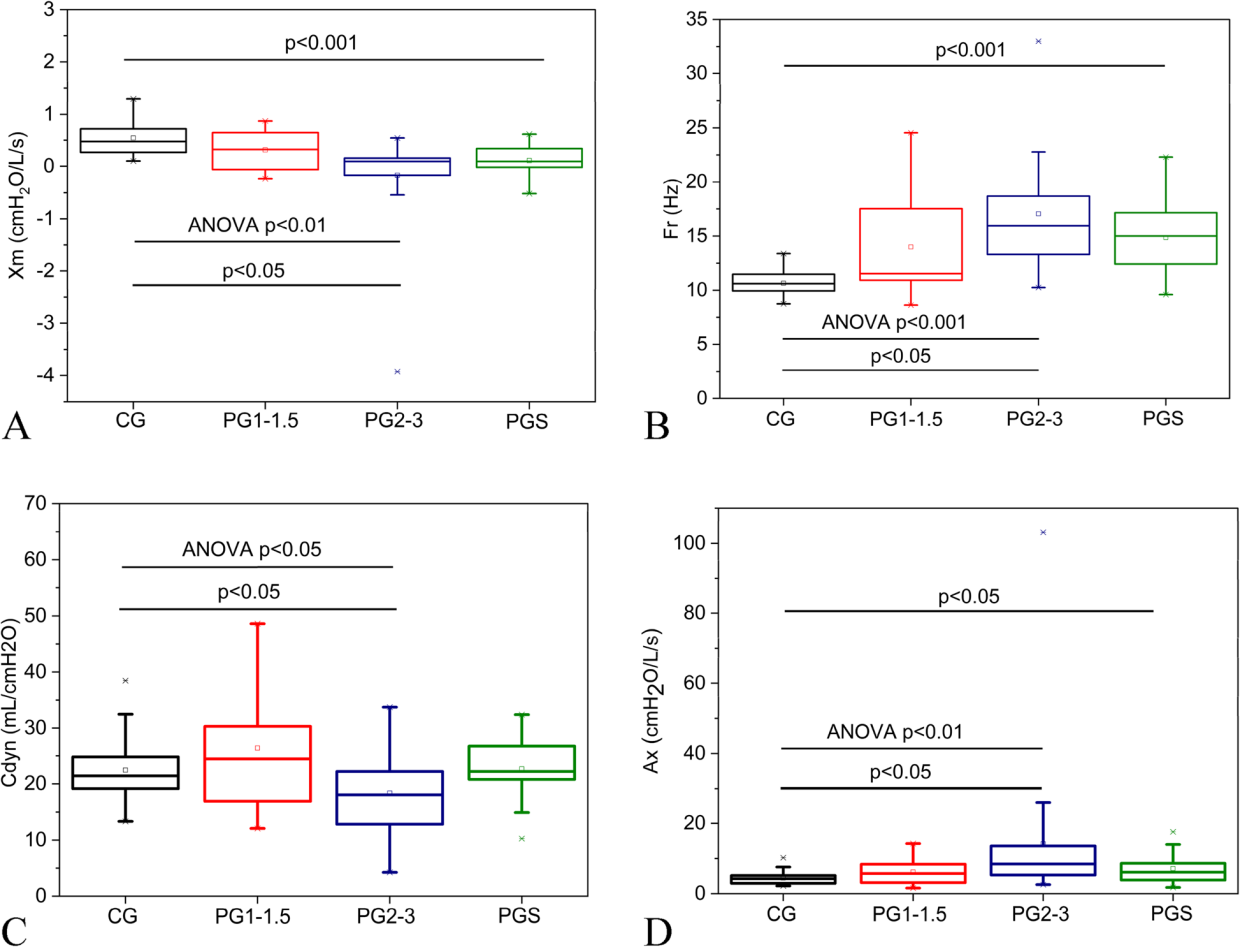
Effect of Parkinson's Disease on the Reactive oscillometric parameters. Xm, mean reactance (**A**); Fr, resonance frequency (**B**); Cdyn, dynamic compliance (**C**); Ax, area under the reactance curve (**D**)

Increasing Hoehn and Yahr Scale introduced significant reductions in Xm (Fig. 3A; ANOVA *p* < 0.01) and Cdyn (Fig. 3C), as well as a significant increase in Fr (Fig. 3B) and Ax (Fig. 3D). Post hoc analysis showed significant changes in patients in the more advanced stage (PG2-3) in comparison with the control group for all of the reactive parameters (*p* < 0.05).

Multiple comparisons among the PGS and the other studied groups revealed a significant reduction in Xm (Fig. 3A) in comparison with the CG (*p* < 0.05). Similar analysis showed a significant increase in Fr (Fig. 3B) and Ax (Fig. 3D).

**Fig. 4:**
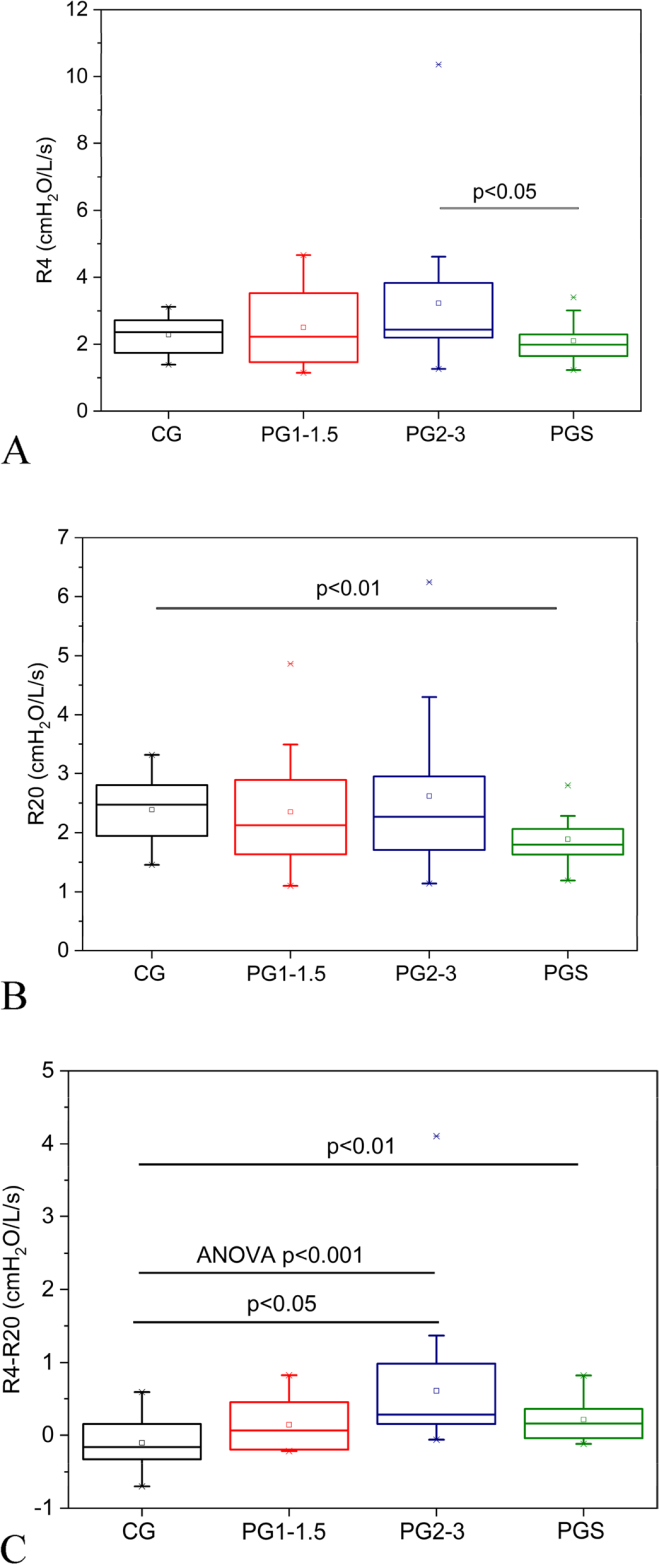
Analysis of variance (ANOVA) describing the effect of Parkinson Disease stage on the resistive parameters. A comparative analysis including the smoking patients and the other studied groups is also described. Only significant values are shown. R4, resistance at 4Hz **A**; R20, resistance at 20Hz **B**; R4-R20, resistance difference **C**. CG, control group; PG1-1.5, Parkinson Disease stages 1 to 1.5; PG2-3 stages 2 to 3; PGS, Parkinson Disease smoking patients

Figure 4 shows that increasing Hoehn and Yahr Scale does not introduce significant changes in R4 and R20 (Figs. 4A and B, respectively) but resulted in a significant increase in R4-R20 (Anova, *p* < 0.001; Fig. 4C). Multiple comparisons in these three groups showed a significant increase in R4-R20 in the more advanced stage in comparison with controls (*p* < 0.05).

The Parkinson's smoking group showed smaller values of R4 in comparison with PG2-3 (*p* < 0.05; Fig. 4A). It was also observed a significant reduction in R20 (*p* < 0.01; Fig. 4B) and increase in R4-R20 (*p* < 0.01; Fig. 4C) in comparison with the control group.

**Fig. 5:**
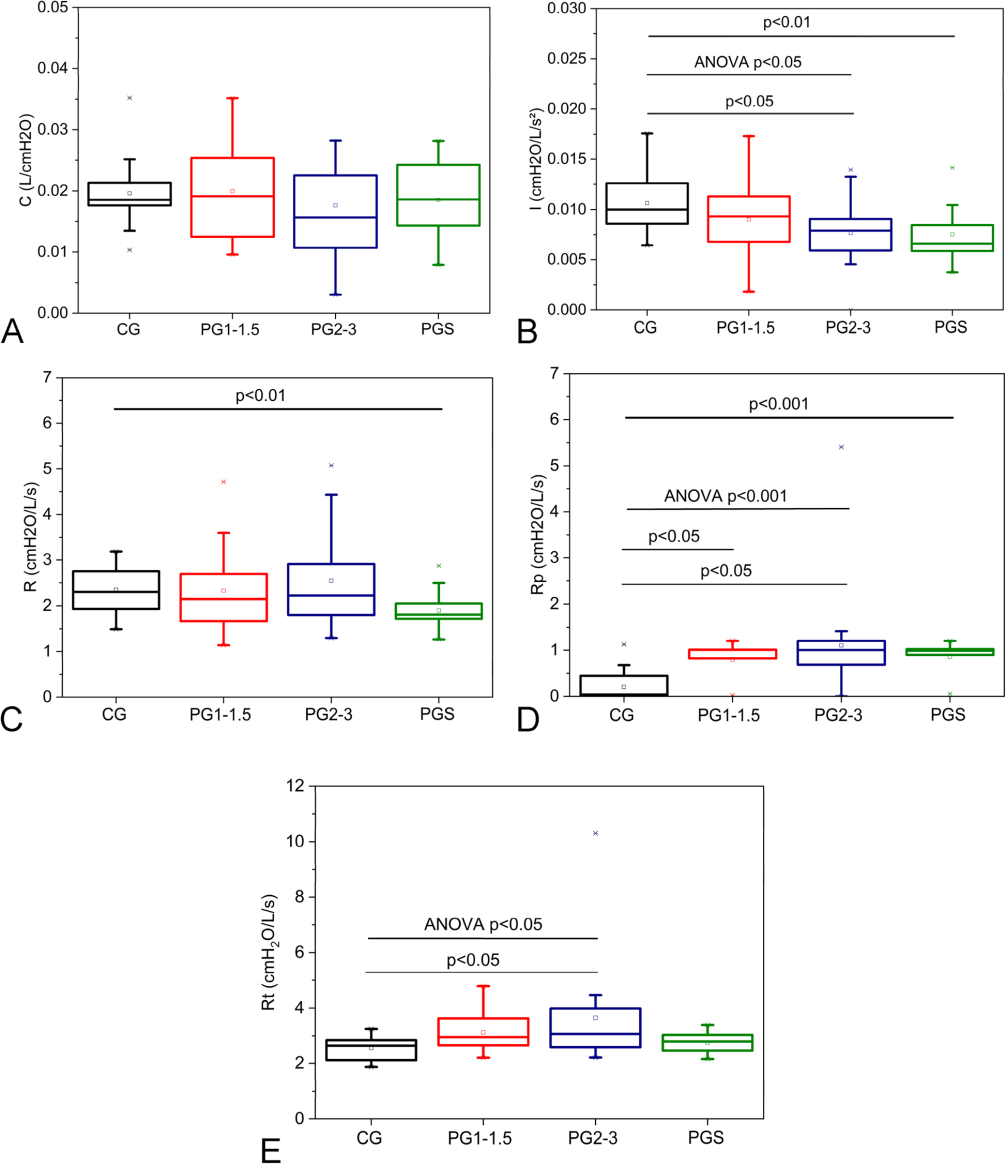
Analysis of variance (ANOVA) describing the effect of Parkinson Disease stage on the parameters estimated in the extended RIC model. A comparative analysis including the smoking patients and the other studied groups is also described. Only significant values are shown. Alveolar compliance (C, A); pulmonary inertance (I, B); central airway resistance (R, C); peripheral resistance (Rp, D) and total resistance (Rt, E). CG, control group; PG1-1.5, Parkinson Disease stages 1 to 1.5; PG2-3 stages 2 to 3; PGS, Parkinson Disease smoking patients

Considering the respiratory eRIC model parameters, C do not showed significant changes (Fig. 5A), while a significant reduction in I (Fig. 5B; *p* < 0.05) was found as PD progressed, in addition to a significant increase in Rp (*p* < 0.001; Fig. 5D) and Rt (*p* < 0.05; Fig. 5E). Post hoc evaluations revealed a significant decrease of the inertance in the PG2-3 in comparison with the CG (*p* < 0.05; Fig. 5B). This analysis also showed that R4-R20 increased in the PG1-1,5 and PG2-3 in comparison with controls (*p* < 0.05; Fig. 5D).

The comparison of the PGtab with the other groups resulted in a significant reduction in I and R as compared with controls (*p* < 0.01; Fig. 5B and C, respectively). In addition, it was also observed a significant increase in Rp (*p* < 0.001; Fig. 5D) in relation to controls.

The correlation analysis (Tables 2 and 3) showed significant direct associations among Xm and I with MEP (R = 0.37, *p* = 0.02; R = 0.34, *p* = 0.03, respectively) and Fr with MIP (R = 0.34, *p* = 0.03). Inverse relationships were observed among Xm and MIP (R = -0.36, *p* = 0.02), I and MIP (R = -0.36, *p* = 0.02), and Rt and MEP (R = -0.34, *p* = 0.03).

**Table 2:** Correlation coefficient (r), coefficient of determination (r^2^) and correlation significance level (p) of respiratory oscillometry in relation to manovacuometry

		**Xm**	**Fr**	**Cdin**	**Ax**	**R4**	**R20**	**R4-R20**
MIP	R r^2^ p	**-0.36** 0.13 **0.02**	**0.34** 0.11 **0.03**	0.0017 0.000002 0.99	0.17 0.02 0.29	0.12 0.01 0.43	0.02 0.0004 0.89	0.23 0.05 0.15
MEP	R r^2^ p	**0.37** 0.13 **0.02**	-0.29 0.08 0.06	0.09 0.009 0.55	-0.30 0.09 0.05	-0.26 0.06 0.10	-0.21 0.04 0.18	-0.26 0.06 0.10

**Table 3:** Correlation coefficient (r), coefficient of determination (r^2^) and correlation significance level (p) of the parameters of the extended RIC model of respiratory oscillometry in relation to manovacuometry

		**C**	**I**	**R**	**Rp**	**Rt**
MIP	R r^2^ p	-0.04 0.001 0.80	**-0.36** 0.13 **0.02**	0.0004 0.0000 0.99	0.30 0.09 0.06	0.15 0.02 0.33
MEP	R r^2^ p	0.14 0.02 0.38	**0.34** 0.12 **0.03**	-0.18 0.03 0.26	-0.20 0.04 0.20	**-0.34** 0.11 **0.03**

ROC analysis considering the CG and PG1–1.5 showed that only Rp presented a good value for clinical use, with AUC = 0.858. Similar analysis considering PG2-3 showed adequate diagnostic accuracy (AUC > 0.80) for Xm, R4-R20, and Rp, while Fr showed high diagnostic accuracy (AUC > 0.948). Changes in smoking patients were adequately identified by Xm, Fr, I, and Rp (AUC > 0.80), where the best parameter was Rp (AUC = 0.896). The reader may find a detailed description of these ROC analyses in the additional online files (please see additional files T1, T2, T3 and F1, F2, F3).

## Discussion

This study evaluated the usefulness of oscillometry in PD patients. Four major findings were obtained: 1) Traditional parameters and the studied eRIC model provided a detailed description of the PD pathophysiology. The initial stages were related to a reduction in ventilation homogeneity associated with changes in peripheral airways. More advanced stages also included a restrictive ventilatory pattern; 2) Changes in oscillometric parameters were correlated with respiratory muscle weakness; 3) eRIC modeling could adequately identify early abnormal changes, and 4) changes in the more advanced stages were diagnosed with high accuracy.

Zhang et al. evaluated volunteers with Hoehn & Yahr = 1 and did not observe a significant reduction in FVC%, FEV1%, and FEV1/FVC% concerning controls [42]. De pandis et al. evaluated individuals with Hoehn and Yahr scores from 3 to 5 in the ON and OFF stages [43] and suggested the presence of a restrictive ventilatory pattern, characterized by a significant reduction in FVC% and FEV1% with normal values of FEV1/FVC%, in the OFF state compared to the ON state. Volunteers up to 3 on the modified Hoehn and Yahr scale were evaluated in the present work (Table 1). Therefore, the lower motor impairment may explain the absence of a significant difference in spirometric parameters in comparing the different groups with PD.

No significant spirometric decline was seen in the PGtab compared to the CG (Table 1). It is worth noting that the mean smoking history of the PGtab was 32.3 pack-years with a standard deviation of 35.5, where a worsening of ventilation would be expected due to moderate exposure to the effects of cigarettes. Such findings were found by Faria et al., who compared healthy individuals with smokers with different degrees of smoking history and found a significant reduction in spirometric parameters as exposure increased [44]. The neuroprotection of smoking related to nicotine, the main component of cigarettes, causing increased release and concentration of dopamine in the striatum in PD is well described in experimental models, including relieving motor symptoms. However, this relationship between symptomatic improvement in humans is still debated in the literature [7, 45]. This relief of motor symptoms in the PGtab could lead to better performance in spirometry, assuming that the ventilatory changes result from these symptoms, which could justify the findings.

The work by Zhang et al. observed a significant reduction in MIP and MEP in patients with Hoehn & Yahr = 1 compared to controls [42], as well as Wang et al., in subjects with Hoehn & Yahr 2 to 5 [46]. In Fig. 2, expiratory muscle weakness was found in all Parkinson's groups, and inspiratory muscle weakness in the group with more significant motor impairment. Although individuals with severe impairment were not studied in the present study, the functional decline is also present in mild and, mainly, moderate stages, characterized by the accentuation of adverse motor symptoms, including gait difficulty. These factors can contribute to a decrease in daily activities, resulting in the deconditioning of the global muscles and, consequently, the weakness of the respiratory muscles [13, 47, 48]. Therefore, deconditioning associated with chest stiffness and bradykinesia may explain these findings.

Only two previous studies used respiratory oscillometry in PD [28, 29]. De Bruin et al. evaluated a group of 10 individuals, including three former smokers and one active smoker, with Hoehn and Yahr scores from 2 to 4, pre and post-apomorphine induction. No significant differences were found in the elastic and resistive properties of pre and post-apomorphine induction [28]. On the other hand, Sampath et al. evaluated two groups of non-smoking patients (Hoehn and Yahr 1 and 2), with 7 individuals each. Significant increases were found according to the progression of PD in resistances (R5 and R20). This finding was attributed to changes in the proximal and distal airways [29]. The present study extends the cited studies, using a larger sample and a more detailed analysis of respiratory mechanics, including respiratory modeling. Additional improvements include a control group and a group of active or former smokers.

Chest stiffness, characteristic of PD, led to a deficit in expansion and increased elastic recoil of the system. This increase in elastic properties explains the observed reductions in Cdyn and Xm and the increase in Fr and Ax according to PD progression observed in Fig. 3. The changes in chest stiffness are not homogeneous, reducing the homogeneity of these elastic properties. The cited parameters also reflected these abnormalities.

In agreement with these findings, Miranda et al. evaluated patients with scleroderma, in which one of the manifestations is a restrictive pattern. A significant reduction in Xm and Cdyn was observed, with a significant increase in Fr in the restrictive group compared to the control [26]. Also in line with these results, Mori et al. evaluated individuals with idiopathic pulmonary fibrosis and observed a significant reduction in reactance at 5 Hz and a significant increase in Fr as the disease progresses [49]. The typical pattern of restrictive ventilatory dysfunction observed in these studies is explained by the increase in elastic recoil due to the involvement of the lung parenchyma generated by fibrosis [26, 49]. In PD, this pattern may be a consequence of the increase in elastic properties of the chest wall. De Troyer et al. suggested the association between respiratory muscle weakness and loss of lung volume and, consequently, restrictive dysfunction when evaluating individuals with neuromuscular diseases [50]. Therefore, the ventilatory muscle weakness detected in the present study (Additional file F1) may also be an essential factor in the observed restrictive changes. The significant correlation between Xm and Fr with muscle performance parameters MIP and MEP supports this hypothesis (Fig. 2).

Smoking patients showed similar abnormalities despite the absence of changes in Cdyn (Fig. 3). Such findings can be explained by the increased elastic recoil and ventilation heterogeneity caused by chest stiffness.

Interestingly, no significant increases were observed as the PD progressed concerning R4 and R20 (Figs. 4A and B, respectively). This indicates the absence of alterations in the more central airways. These results are in contrast to that obtained by Sampath et al.[32], which found significant increase according to disease progression in R5 and R20. These discrepancies may be explained, at least in part, because the cited study was conducted using an impulse oscillation system (IOS), while our study was performed using the forced oscillation technique (FOT). The IOS yields respiratory system resistance values similar, but not identical to those provided by the classical FOT [50]. These discrepancies may also be related with differences in the studied populations.

The increase in R4-R20 (Fig. 4C) indicates a reduction in ventilation homogeneity in addition to possible changes in peripheral airwaysresistances. Gochicoa-Rangel et al. hypothesized when evaluating children with Duchenne Muscular Dystrophy that the ventilatory muscle weakness characteristic of the disease and causing the restrictive ventilatory pattern may lead to lower lung volumes during ventilation, reducing the airway caliber and increasing resistance [51]. A recent international review on respiratory oscillometry suggests that increased elastic properties can reduce lung volume in an underlying restrictive disease, causing increased airway resistance [27]. The reduction in lung volume due to increased elastic properties and restrictive patterns according to the progression of PD may lead to a lowerair volume in some points of the airways. These abnormalities are reflected in the findings in R4-R20. It is worth noting that the ventilatory muscle weakness found in our sample may also contribute secondarily to lung volume reduction and with the findings in R4-R20.

Using the forced oscillation technique, Ribeiro et al. [23] evaluated the early effects of smoking and COPD. In the smoking group, with a smoking history of 20 pack-years, significant increases in resistance were observed concerning controls [23]. Given our sample's moderate smoking history, we expected to find effects similar to Ribeiro et al. [23]. The role of nicotine in relieving motor symptoms is currently being discussed, and its association with symptomatic improvement in humans is still under debate [7, 45]. Assuming that the respiratory changes result from these symptoms, it is reasonable to hypothesize that this relief could contribute to the absence of changes in R4 (Fig. 4A) and the reduced values of R20 (Fig. 4B).

The increasing in Rp (Fig. 5D) is related to ventilation inhomogeneity. The observed inhomogeneity may result from increased resistance in the distal airways. Gochicoa-Rangel et al. suggested that lower lung volumes during ventilation may reduce airway caliber, increasing airway resistance [51]. Chest stiffness can act similarly, increasing the resistance of the distal airways. It could explain the moderate diagnostic accuracy observed in Rp in the three Parkinson's groups. The increase in peripheral resistances may also explain the increase in Rt with disease progression (Fig. 5E). The absence of changes in R4, R20, and R (Figs. 4A, 4B, and 5C, respectively) provide additional evidence that the changes in PD are mainly related to peripheral airways.

Inertance describes the inertia of the gas mass moved during spontaneous ventilation. A restrictive pattern causes less air to move in the airways during ventilation, explaining the reductions I observed in the comparisons between the different groups with PD and when comparing the PGtab to the CG (Fig. 5B).

The observed correlations among oscillometric parameters and respiratory pressures (Table 2) can be explained by the relationship described by De Troyer et al. These authors observed associations between respiratory muscle weakness and loss of lung volume, which resulted in increased reactance according to the loss of ventilatory muscle strength [50]. Inertance reflects the mass of gas moved during spontaneous ventilation, so the lower the MIP and MEP, the smaller the amount of air moved and, consequently, the lower the inertance. A reasonable inverse correlation was observed between Rt and MEP (Table 3), which can be explained by the deficit in the ventilatory muscles and the resulting increased resistance [51]. These results show that respiratory oscillometry parameters are related to the loss of ventilatory muscle strength and the decline of respiratory mechanics in PD.

Traditional pulmonary function exams are based on maximal effort maneuvers that may be difficult in patients with PD [13]. Respiratory oscillometry is simpler to understand and does not demand complicated respiratory motor control maneuvers. In this context, respiratory oscillometry may provide detailed information on respiratory abnormalities in patients with PD, demanding a simple exam. Considering the diagnostic use of this method, even in the initial stages (H&Y stage 1–1.5), Rp presented high sensibility, showing an adequate value for clinical use (Additional Table T1, Additional Figure F1). This finding agrees with the hypothesis that peripheral resistance is the initial site of changes in PD patients. However, previous studies have shown that FEV1 remains unchanged until 75% of the small airways are obstructed [51, 52]. Therefore, these results provide evidence that oscillometry may complement spirometric exams in patients with PD.

As expected, the diagnostic accuracy increased in patients with advanced stages (H&Y stage 2–3), in which Fr achieved high accuracy (Additional Table T2, Additional Figure F2). The increase in elasticity and the reduction in ventilation homogeneity may explain this finding.

It was pointed out previously that oscillometry is highly sensitive to changes in respiratory mechanics [53, 54]. In close agreement with this hypothesis, respiratory abnormalities in smoking patients were adequately identified by several parameters (Additional Table T3, Additional Figure F3), providing clear evidence that oscillometry may help analyze these patients. It is worth noting that the highest diagnostic accuracy was obtained from Rp, obtained using the eRIC model.These results are consistent with recent studies using this model to investigate peripheral resistance in childhoodcystic fibrosis [26] and preschool asthma[46].

The high prevalence of cognitive and functional impairment in patients with PD introduces many difficulties in performing spirometry and plethysmography exams. These practical limitations highlight the clinical importance of the respiratory oscillometry, which allowed a simplified analysis in this class of volunteers who were unable to perform complex respiratory maneuvers.

It was recently pointed out that clinical application of oscillometry in restrictive lung disease is less established in comparison to obstructive disease [25]. Reactive parameters providing clear information concerning restrictive abnormalities were described by our group [55–59] and other researchers [60–63]. In addition, a recent study showed a clear differentiation between asthma and restrictive respiratory diseases using Fr [64]. We need to be cautious with this important point, and more studies are necessary to reach a consensus that oscillometry is able of detecting restrictive changes. The present study is a contribution in this direction.

Although strict criteria were adopted for the design of this study, the findings are subject to three limitations. First, patients were not tested with plethysmography because of little cooperation. The findings were not correlated with functional tests, which would allow a global assessment of pulmonary limitation associated with impairment of activities of daily living. Second, volunteers with PD could have been divided into dominant tremors and rigidity to assess the specific effect of these symptoms on lung function. Third, the smoking group could have been divided based on their motor impairment, helping to assess the effects of smoking, although this was not the focus of this work. Such limitations can be taken as suggestions for continuing the research.

## Conclusions

The initial stages of PD are related to a reduction in ventilation homogeneity and possible changes in peripheral airwayresistances. More advanced stages also include a restrictive ventilatory pattern. These changes were correlated with respiratory muscle weakness and were observed in mild and moderate stages of PD in smokers and non-smokers. Oscillometric parameters are able to identify respiratory changes in the early stages of PD and obtain high diagnostic accuracy in more advanced stages of the disease.

### Supplementary Information

**Additional file 1:** **Figure F1.** Receiver Operator Characteristic curve of the most discriminating parameter between the CG and PG1–1.5

**Additional file 2:** **Figure F2.** Receiver Operator Characteristic curve of the most discriminating parameters between the CG and PG2–3. Resonance frequency (A) and peripheral resistance (B).

**Additional file 3:** **Figure F3.** Receiver Operator Characteristic curve of the most discriminating parameters between the CG and smoking patients. Resonance frequency (A) and peripheral resistance (B).

**Additional file 4:** **Table T1.** Values of area under the curve (AUC), sensitivity (Se), specificity (Sp) and cut-off points for traditional parameters and eRIC model in patients with Parkinson 1–1.5. Adequate diagnostic accuracy (AUC >0.80) are indicated in bold.

**Additional file 5:** **Table T2.** Values of area under the curve (AUC), sensitivity (Se), specificity (Sp) and cut-off points for traditional parameters and eRIC model in patients with Parkinson 2–3. Adequate diagnostic accuracy (AUC >0.80) are indicated in bold.

**Additional file 6:** **Table T3.** Values of area under the curve (AUC), sensitivity (Se), specificity (Sp) and cut-off points for traditional parameters and eRIC model in patients with Parkinson smoker. Adequate diagnostic accuracy (AUC >0.80) are indicated in bold.

## Data Availability

The dataset supporting the conclusions of this article will be available in the Open Science Framework repository at the following link: https://osf.io/c34yd/?view_only=42108db8983e4b11ac64b346853e0376
