# Supplementary material for: Oscillometry of the respiratory system in Parkinson's disease: physiological changes and diagnostic use
Source: BMC Pulm Med. 2023 Oct 26;23:406. doi: 10.1186/s12890-023-02716-w (PMC10605979; doi:10.1186/s12890-023-02716-w)
Supplement: Supplementary file 1 — Additional file 1: Figure F1. Receiver Operator Characteristic curve of the most discriminating parameter between the CG and PG1–1.5 [file 12890_2023_2716_MOESM1_ESM.docx]

Specificity

Sensibility

Figure F1 – Receiver Operator Characteristic curve of the most discriminating parameter between the CG and PG1–1.5
