# Supplementary material for: Oscillometry of the respiratory system in Parkinson's disease: physiological changes and diagnostic use
Source: BMC Pulm Med. 2023 Oct 26;23:406. doi: 10.1186/s12890-023-02716-w (PMC10605979; doi:10.1186/s12890-023-02716-w)
Supplement: Supplementary file 2 — Additional file 2: Figure F2. Receiver Operator Characteristic curve of the most discriminating parameters between the CG and PG2–3. Resonance frequency (A) and peripheral resistance (B). [file 12890_2023_2716_MOESM2_ESM.docx]

A

Specificity

Sensibility

B

Specificity

Sensibility

Figure F2 – Receiver Operator Characteristic curve of the most discriminating parameters between the CG and PG2–3. Resonance frequency (A) and peripheral resistance (B).
