# Supplementary material for: Oscillometry of the respiratory system in Parkinson's disease: physiological changes and diagnostic use
Source: BMC Pulm Med. 2023 Oct 26;23:406. doi: 10.1186/s12890-023-02716-w (PMC10605979; doi:10.1186/s12890-023-02716-w)
Supplement: Supplementary file 4 — Additional file 4: Table T1. Values of area under the curve (AUC), sensitivity (Se), specificity (Sp) and cut-off points for traditional parameters and eRIC model in patients with Parkinson 1–1.5. Adequate diagnostic accuracy (AUC >0.80) are indicated in bold. [file 12890_2023_2716_MOESM4_ESM.docx]

Table T1

Values of area under the curve (AUC), sensitivity (Se), specificity (Sp) and cut-off points for traditional parameters and eRIC model in patients with Parkinson 1–1.5. Adequate diagnostic accuracy (AUC >0.80) are indicated in bold.

|  | AUC | 95% IC | Se (%) | Sp (%) | Cut-off |  |
| --- | --- | --- | --- | --- | --- | --- |
| Traditional |  |  |  |  |  |  |
| Xm | | 0.627 | 0.442 – 0.788 | 38.46 | 95.00 | 0.1143 |
| Fr | | 0.738 | 0.557 – 0.875 | 84.62 | 55.00 | 10.76953 |
| Cdyn | | 0.569 | 0.386 – 0.740 | 61.54 | 70.00 | 23.52608 |
| Ax | | 0.615 | 0.431 – 0.779 | 53.85 | 90.00 | 5.68993 |
| R4 | 0.523 | 0.343 – 0.699 | 38.46 | 95.00 | 3.01703 |  |
| R20 | 0.573 | 0.390 – 0.743 | 46.15 | 85.00 | 1.85589 |  |
| R4-R20 | 0.685 | 0.500 – 0.835 | 100.00 | 40.00 | 0.26615 |  |
| eRIC model |  |  |  |  |  |  |
| C | 0.500 | 0.322 – 0.678 | 61.54 | 5.00 | 0.01286 |  |
| I | 0.612 | 0.427 – 0.776 | 46.15 | 80.00 | 0.00766 |  |
| R | 0.588 | 0.404 – 0.756 | 38.46 | 90.00 | 1.74236 |  |
| Rp | **0.858** | 0.692 – 0.954 | 76.92 | 95.00 | 0.67556 |  |
| Rt | 0.731 | 0.548 – 0.870 | 53.85 | 85.00 | 2.89559 |  |
