# Supplementary material for: Oscillometry of the respiratory system in Parkinson's disease: physiological changes and diagnostic use
Source: BMC Pulm Med. 2023 Oct 26;23:406. doi: 10.1186/s12890-023-02716-w (PMC10605979; doi:10.1186/s12890-023-02716-w)
Supplement: Supplementary file 5 — Additional file 5: Table T2. Values of area under the curve (AUC), sensitivity (Se), specificity (Sp) and cut-off points for traditional parameters and eRIC model in patients with Parkinson 2–3. Adequate diagnostic accuracy (AUC >0.80) are indicated in bold. [file 12890_2023_2716_MOESM5_ESM.docx]

Table T2

Values of area under the curve (AUC), sensitivity (Se), specificity (Sp) and cut-off points for traditional parameters and eRIC model in patients with Parkinson 2–3. Adequate diagnostic accuracy (AUC >0.80) are indicated in bold.

|  | AUC | 95% IC | Se (%) | Sp (%) | Cut-off |  |
| --- | --- | --- | --- | --- | --- | --- |
| Traditional |  |  |  |  |  |  |
| Xm | | **0.893** | 0.756 – 0.968 | 80.95 | 90.00 | 0.20283 |
| Fr | | **0.948** | 0.830 – 0.993 | 90.48 | 95.00 | 12.12288 |
| Cdyn | | 0.700 | 0.537 – 0.833 | 66.67 | 80.00 | 18.90624 |
| Ax | | 0.783 | 0.627 – 0.896 | 71.43 | 90.00 | 5.68993 |
| R4 | 0.626 | 0.461 – 0.772 | 38.10 | 100.00 | 3.11816 |  |
| R20 | 0.533 | 0.371 – 0.690 | 19.05 | 100.00 | 1.4376 |  |
| R4-R20 | **0.838** | 0.690 – 0.934 | 85.71 | 75.00 | 0.10184 |  |
| eRIC model |  |  |  |  |  |  |
| C | 0.698 | 0.534 – 0.831 | 52.38 | 95.00 | 0.01339 |  |
| I | 0.767 | 0.609 – 0.884 | 66.67 | 80.00 | 0.00821 |  |
| R | 0.505 | 0.344 – 0.664 | 28.57 | 85.00 | 1.80101 |  |
| Rp | **0.864** | 0.721 – 0.951 | 76.19 | 95.00 | 0.67556 |  |
| Rt | 0.750 | 0.590 – 0.872 | 61.90 | 85.00 | 2.89559 |  |
