# Supplementary material for: Oscillometry of the respiratory system in Parkinson's disease: physiological changes and diagnostic use
Source: BMC Pulm Med. 2023 Oct 26;23:406. doi: 10.1186/s12890-023-02716-w (PMC10605979; doi:10.1186/s12890-023-02716-w)
Supplement: Supplementary file 6 — Additional file 6: Table T3. Values of area under the curve (AUC), sensitivity (Se), specificity (Sp) and cut-off points for traditional parameters and eRIC model in patients with Parkinson smoker. Adequate diagnostic accuracy (AUC >0.80) are indicated in bold. [file 12890_2023_2716_MOESM6_ESM.docx]

Table T3

Values of area under the curve (AUC), sensitivity (Se), specificity (Sp) and cut-off points for traditional parameters and eRIC model in patients with Parkinson smoker. Adequate diagnostic accuracy (AUC >0.80) are indicated in bold.

|  | AUC | 95% IC | Se (%) | Sp (%) | Cut-off |  |
| --- | --- | --- | --- | --- | --- | --- |
| Traditional |  |  |  |  |  |  |
| Xm | | **0.842** | 0.674 – 0.945 | 69.23 | 90.00 | 0.19959 |
| Fr | | **0.885** | 0.725 – 0.969 | 84.62 | 90.00 | 11.66993 |
| Cdyn | | 0.550 | 0.368 – 0.723 | 76.92 | 50.00 | 20.73443 |
| Ax | | 0.712 | 0.528 – 0.855 | 53.85 | 90.00 | 5.68993 |
| R4 | 0.600 | 0.415 – 0.766 | 76.92 | 55.00 | 2.29084 |  |
| R20 | 0.773 | 0.594 – 0.900 | 76.92 | 70.00 | 2.06573 |  |
| R4-R20 | 0.758 | 0.578 – 0.889 | 100.00 | 55.00 | -0.1459 |  |
| eRIC model |  |  |  |  |  |  |
| C | 0.535 | 0.353 – 0.709 | 46.15 | 90.00 | 0.01525 |  |
| I | **0.808** | 0.633 – 0.923 | 76.92 | 80.00 | 0.00846 |  |
| R | 0.750 | 0.569 – 0.884 | 61.54 | 85.00 | 1.82788 |  |
| Rp | **0.896** | 0.740 – 0.975 | 76.92 | 95.00 | 0.67556 |  |
| Rt | 0.627 | 0.442 – 0.788 | 46.15 | 80.00 | 2.85233 |  |
